# Effects of Exercise and Physical Activity on Anxiety

**DOI:** 10.3389/fpsyt.2013.00027

**Published:** 2013-04-23

**Authors:** Elizabeth Anderson, Geetha Shivakumar

**Affiliations:** ^1^VA North Texas Health Care SystemDallas, TX, USA; ^2^Department of Psychiatry, University of Texas Southwestern Medical CenterDallas, TX, USA

## Introduction

The beneficial effects of regular physical activity on health are indisputable in the field of modern medicine. Exercise is often the first step in lifestyle modifications for the prevention and management of chronic diseases. According to a US Department of Health and Human Services report on physical activity, regular exercise significantly reduced causes of mortality by up to 30% for men and women (DHHS, 2002). These health benefits are seen consistently across all age groups and racial/ethnic categories. The Centers for Disease Control and Prevention currently recommends 30 min of moderate- to high-intensity exercise for at least 5 days a week for all healthy individuals (DHHS, 2002).

In addition to significantly lowering causes of mortality, regular exercise and physical activity lowers prevalence of chronic disease(s). There is a strong evidence to support that 2–2.5 h of moderate- to high-intensity exercise per week is sufficient to reduce one’s risk for the occurrence of a chronic disease(s). Numerous epidemiological studies have shown that exercise improves one’s self-esteem, and a sense of wellbeing. Individuals who exercise regularly exhibit slower rates of age-related memory and cognitive decline in comparison to those who are more sedentary. Such observations have provided the basis for using exercise to improve memory and cognition in cognitive disorders such as Alzheimer’s Dementia. Adults who engage in regular physical activity experience fewer depressive and anxiety symptoms, thus supporting the notion that exercise offers a protective effect against the development of mental disorders (van Minnen et al., 2010).

Anxiety disorders are common psychiatric conditions with a lifetime prevalence of nearly 29% in the United States (Kessler et al., 2005). These disorders are chronic, debilitating, and impact multiple aspects of one’s life. The economic burden of anxiety disorders in the US was estimated to be $42.3 billion in the 1990s (Greenberg et al., 1999). The prominent anxiety disorders defined in the Diagnostic and Statistical Manual of Mental Disorders (DSM-IV) are General Anxiety Disorder (GAD), Panic Disorder (PD), Posttraumatic Stress Disorder (PTSD), Obsessive Compulsive Disorder (OCD), Social Anxiety Disorder, and Specific Phobia (APA, 2000). The exact etiology and pathophysiology of these conditions is not fully understood. Comprehending the effects of exercise and physical activity on the mechanisms of anxiety disorders might further our knowledge of these psychiatric disorders. The purpose of this article is to highlight the known and emerging mechanisms that may result in the anxiolytic effects of exercise.

## Physiological Mechanisms

Broadly, regular exercise results in physiological changes and adaptations in the human body. Studies have shown that regular aerobic exercise is associated with lower sympathetic nervous system and hypothalamic-pituitary-adrenal (HPA) axis reactivity (Crews and Landers, 1987; Åstrand, 2003; Jackson and Dishman, 2006; Rimmele et al., 2007).

## Hypothalamic-Pituitary-Adrenal Axis

The HPA axis plays a critical role in developing adaptive responses to physical and psychological stressors (De Kloet et al., 2005). Dysregulations in the HPA axis have long been implicated in the manifestations of depressive and anxiety symptoms (Landgraf et al., 1999; Steckler et al., 1999). Acute stress leads to alterations in adrenocorticotropic hormone (ACTH) and excess levels of glucocorticoids. Chronic stress, as seen in PTSD, has been associated with lower concentrations of peripheral cortisol and upregulation of the glucocorticoid receptors resulting in increased central feedback sensitivity. Depending on the experimental paradigm used for chronic stress, some studies have shown decreased plasma ACTH and corticosterone levels while other studies have shown increased corticosterone secretion (Irwin et al., 1986; Kant et al., 1987). In preclinical studies, voluntary exercise alters the releases of corticotrophin-releasing factor (CRF) from the hypothalamus and ACTH from the anterior pituitary (Salmon, 2001; Droste et al., 2003). These findings suggest that exercise induced changes in the HPA axis modulates stress reactivity and anxiety in humans.

## Monoamine System

Abnormalities in monoamine function in the brain have been implicated in the pathophysiology of anxiety spectrum disorders. In animal studies, learned helplessness resulting from chronic electric shock was associated with a reduced release of serotonin in the frontal cortex (Miller et al., 1975; Petty et al., 1992). Learned helplessness is also associated with a depletion of norepinephrine (Petty et al., 1993). It is postulated that the reductions in serotonergic and noradrenergic levels reflects synthesis not being able to keep up with demand (Charney et al., 2004). Animal models also provide evidence that regular aerobic exercise increases serotonergic and noradrenergic levels in the brain, similar to the effects of antidepressants (Praag, 1982; Veale, 1987; Chaouloff, 1989; Meeusen and De Meirleir, 1995). Researchers have observed increased extraneuronal uptake of norepinephrine and increased levels of norepinephrine in the hippocampus and frontal cortex of rodents after treadmill training and wheel running (Dunn et al., 1996; Dishman, 1997). Increases in serotonin synthesis, metabolism, and release have been noted following exercise (Dunn and Dishman, 1991; Meeusen and De Meirleir, 1995; Wilson and Marsden, 1996; Chaouloff, 1997). Animal models utilizing chronic voluntary wheel running have also shown small increases in serotonergic neural activity in the dorsal raphe nucleus, an area of brain that is abundant in serotonergic neurons, during uncontrollable stress (Greenwood et al., 2003). Treadmill exercise training also increases levels of prepro-galanin mRNA, suggesting that gene expression for galanin is sensitive to the stress from exercise training and may have a “neuromodulating role” in the noradrenergic response in the locus ceruleus, an area of brain rich in noradrenergic neurons (O’Neal et al., 2001).

## Opioid System

Another possible mechanism for the anxiolytic effects of exercise is via mediation by the endogenous opioid system. Endogenous opioids have a role in the regulation of mood and emotional responses (Bodnar and Klein, 2005). For example, abnormal levels of both central and peripheral β-endorphins have been discovered in individuals diagnosed with depression (Scarone et al., 1990; Darko et al., 1992). The endorphin hypothesis posits that the mood elevations and reduced anxiety following acute exercise is due to the release and binding of β-endorphins (endogenous opioids) to their receptor sites in the brain. Studies demonstrate that exercise increases endogenous opioid activity in the central and peripheral nervous system and may induce a euphoric state and reduce pain (Harber and Sutton, 1984; Morgan, 1985; North et al., 1990; Thorén et al., 1990). When opioid antagonists were administered following regular exercise, the endorphin produced analgesic effects were attenuated, but there were no changes in the mental health benefits suggesting that the exercise-related surge in endorphins may not completely account for mental health benefits in these studies (Carr et al., 1981; Moore, 1982; Howlett et al., 1984; Thorén et al., 1990; Yeung, 1996).

## Neurotropic Factors

Brain-derived neurotrophic factor (BDNF), the most abundant neurotrophin in the brain has been linked to both anxiety and depression. Stress-induced depressive and anxious behaviors are correlated with decreased BDNF levels especially in the hippocampus (Duman and Monteggia, 2006). Furthermore, infusions of BDNF into the dorsal raphe nucleus have been shown to have an a antidepressant effect (Altar, 1999). Evidence also suggests that BDNF may be a mediator of the anxiety reducing effects of antidepressant medications (Chen et al., 2006). Increases in BDNF following physical activity have also been observed. Following 20 days of voluntary wheel running compared to non-wheel running rats, BDNF mRNA levels increased in the hippocampus and caudal neocortex (Meeusen and De Meirleir, 1995; Russo-Neustadt et al., 1999). These changes in BDNF increases functioning in the serotonergic system and may promote neuronal growth (Altar, 1999).

## Evidence for Neurogenesis

New neuronal growth in the adult brain, particularly in the hippocampus, has been implicated in the treatment of psychiatric conditions including depression and anxiety (Eisch, 2002). Detection and evaluation of hippocampal neurogenesis is an active area of investigation in recent years (Eisch, 2002). In primate models of chronic stress, the hippocampus has been shown to be highly sensitive to the toxic effects of excessive glucocorticoids, thus impairing the process of neurogenesis (Uno et al., 1989). Neuroplasticity is further supported by the stress-related changes found in studies of hippocampus function. Animal studies have shown exercise up regulates hippocampal neurogenesis (Duman et al., 2001). Exercise is also believed to positively influence surrogate measures of adult hippocampal neurogenesis such as β-endorphins, vascular endothelial growth factor, BDNF, and serotonin, all of which are thought be common pathophysiologic mechanisms for anxiety disorders.

## Psychological Mechanisms

### Anxiety sensitivity and exposure

Anxiety sensitivity is a term for the tendency to misinterpret and catastrophize anxiety-related sensations based on the belief that they will result in disastrous physical, psychological, and/or social outcomes (Broman-Fulks and Storey, 2008; Smits et al., 2008). McWilliams and Asmundson (2001) found an inverse relationship between anxiety sensitivity and exercise frequency and suggested that this relationship was due to avoidance of the physiological sensations of exercise that may be interpreted as anxiety and panic. A number of research studies have pointed to the effectiveness of short-term aerobic exercise to reduce anxiety sensitivity (Broman-Fulks and Storey, 2008; Smits et al., 2008; Ströhle et al., 2009). Exposing someone with high anxiety sensitivity to the physiological symptoms they fear, such as rapid heartbeat, in the context of physical exercise increases their tolerance for such symptoms (McWilliams and Asmundson, 2001). This exposure reveals that the feared physiological sensations may be uncomfortable, but do not pose a serious threat (Ströhle et al., 2009). Repeated exposures through regular aerobic exercise may also facilitate habituation to the feared sensations (Beck and Shipherd, 1997).

### Self-efficacy

According to social cognitive theory, one’s sense of self-efficacy regarding their ability to exert control over potential threats has an important relationship to anxiety arousal. Individuals who trust their ability to manage potential threats (high self-efficacy) are not plagued by thoughts of worry and experience lower levels of anxiety arousal. Based on the theory of self-efficacy, Bandura posited that a treatment will be successful if it is able to rebuild a sense of self-efficacy by supplying experiences of self-mastery. It has been debated that exercise can increase self-efficacy by supplying experiences of successfully coping with the stress of exercising (Petruzzello et al., 1991). As fitness improves, the individual receives feedback of greater endurance, less pain, greater duration capabilities, etc. As a result, self-efficacy should increase (Petruzzello et al., 1991). In fact, one study suggested that exercise with an emphasis on increasing self-efficacy, in this case, martial arts, was more effective in reducing state anxiety than exercise such as riding a stationary bike (Bodin and Martinsen, 2004). In a study examining the relationship between exercise intensity and self-efficacy effects on anxiety reduction in a non-clinical population, researchers found that the influence of self-efficacy on decreased anxiety was exhibited in the moderate intensity exercise group, but not in the light- and high-intensity exercise groups (Katula et al., 1999). These two studies suggest that exercise providing an optimal level of challenge best utilizes the power of self-efficacy.

### Distraction

Distraction or “time out” has been proposed as another reason why exercise is effective at reducing anxiety. Based on their study that found that distraction techniques such as meditation, and quiet rest were as effective as a single session of exercise in reducing state anxiety, Bahrke and Morgan (1978) suggested that the anxiolytic benefits of exercise may result from it being a distraction from stressors and a “time out” from daily activities. The results of meta-analyses supporting this hypothesis are mixed. Exercise and cognitively based distraction techniques were shown to have equal effectiveness at reducing state anxiety, however exercise was more effective in reducing trait anxiety (Petruzzello et al., 1991). In addition, the anxiolytic effects of exercise have been shown to last for a longer period of time than those produced by therapies based on distraction techniques (Raglin and Morgan, 1985).

## Conclusion

There is strong evidence from animal studies that exercise and regular activity positively impacts the pathophysiological processes of anxiety. Numerous studies and meta-analyses show that exercise is also associated with reduced anxiety in clinical settings. Similar to the heterogenic nature of the anxiety, no single mechanism sufficiently accounts for the anxiolytic nature of exercise. Physical activity positively impacts a number of biological, as well as psychological, mechanisms. The role of exercise in the enhancement of neurogenesis in humans has drawn significant attention in recent years and its implications for anxiety disorders are an exciting area of investigation. Future studies are needed to further this type of work, as well as studies specifically exploring clinical applications of exercise in anxiety disorders.

## Conflict of Interest Statement

The authors declare that research was conducted in the absence of any commercial or financial relationships that could be construed as a potential conflict of interest.
